# Community Outpatient Psychotherapy Engagement Service for Self-harm (COPESS): a feasibility trial protocol

**DOI:** 10.1186/s40814-021-00902-3

**Published:** 2021-08-27

**Authors:** Pooja Saini, Anna Hunt, Peter Taylor, Catherine Mills, Caroline Clements, Helen Mulholland, Cecil Kullu, Mark Hann, Rui Duarte, Felicity Mattocks, Else Guthrie, Mark Gabbay

**Affiliations:** 1 School of Psychology, Liverpool John Moores University, Byrom Street, Liverpool, L3 3AF UK; 2 Division of Psychology & Mental Health, Academic Health Sciences Centre, The University of Manchester, Oxford Road, Manchester, M13 9PL UK; 3 Mersey Care NHS Foundation Trust, V7 Building, Kings Business Park, Prescot, L34 1PJ UK; 4 Department of Primary Care and Mental Health, The University of Liverpool, Waterhouse Building Block B, Liverpool, L69 3GF UK; 5 Biostatistics, Division of Population Health, Health Services Research & Primary Care, Manchester, UK; 6 Penny Lane Surgery, 7 Smithdown Place, Liverpool, L15 9EH UK; 7 School of Medicine, The University of Leeds, Woodhouse, Leeds, LS2 9JT UK

## Abstract

**Background:**

People who self-harm are at high risk for future suicide and often suffer considerable emotional distress. Depression is common among people who self-harm and may be an underlying driver of self-harm behaviour. Self-harm is often repeated, and risk of repetition is highest immediately after an act of self-harm. Readily accessible brief talking therapies show promise in helping people who self-harm, but further evaluation of these approaches is needed. A brief talking therapy intervention for depression and self-harm has been designed for use in a community setting. This mixed methods feasibility study with repeated measures will examine the feasibility and acceptability of the Community Outpatient Psychological Engagement Service for Self-Harm (COPESS) for people with self-harm and depression in the community, compared to routine care.

**Methods:**

Sixty participants with a history of self-harm within the last six months, who are also currently depressed, will be recruited to take part in a feasibility single-blind randomised controlled trial (RCT). Participants will be randomised 1:1 to receive COPESS plus treatment as usual (TAU) or TAU alone. Recruitment will be via General Practitioners (GP) and self-referral. Assessment of feasibility and acceptability will be assessed via quantitative and qualitative methods including measures of recruitment and retention to the feasibility trial, participants’ experience of therapy, completion/completeness of outcome measures at relevant time-points and completion of a service use questionnaire.

**Discussion:**

The results will indicate whether it is feasible to conduct a definitive full trial to determine whether COPESS is a clinically and cost effective intervention for people who self-harm in the community. Qualitative and quantitative data will in addition help identify potential strengths and/or challenges of implementing brief community-based interventions for people who self-harm.

**Trial registration:**

NCT04191122 registered 9th December 2019.

## Background

Self-harm is a public health priority and people with a history of self-harm feature in the Suicide Prevention Strategy for England as a priority group [1–3]. Self-harm defined as *any intentional act of self-injury or self-poisoning regardless of motivation or suicidal intent* [4] is often a sign of underlying distress, predictive of psychological problems and associated with premature death by suicide and other causes [5–9]. In England, there are over 200,000 self-harm presentations to hospital emergency departments (ED) annually [10–12]. Rates of self-harm in primary care are estimated to be double the rates of hospital admissions and the prevalence of self-harm in the community is substantially higher [13–16].

General Practitioners are often the first point of contact for mental health issues in the community, but report feeling under skilled in relation to managing self-harm [17, 18]. National policy and guidance [4] emphasises the need for rapid access to community-based services for self-harm, but referral pathways and treatment options are often unclear to patients and health professionals alike [19].

Symptoms of depression are common among people who self-harm and are thought to be related to the initial occurrence and subsequent repetition of self-harm [20, 21]. However, talking therapies designed for treating depression do not necessarily reduce or improve self-harm-related outcomes [22]. It has been argued that self-harm interventions need to be specifically developed for this context [23, 24].

Talking therapies that target the psychological processes underlying self-harm have been shown to reduce repetition of self-harm [25–27] and depressive symptoms [27, 28], and there is some evidence that cognitive behavioural therapy (CBT) and psychodynamic interpersonal therapy (PIT) were effective in reducing some self-harm behaviours in the short-term [28]. The Cochrane review [25] makes a distinction between brief therapies, usually provided for people who present following self-harm in acute distress, and higher-intensity therapies (such as Dialectical Behavioural Therapy [29]) which are designed to help people who struggle with self-harm and multiple co-existing life problems. The evidence base around brief therapies that benefit people with recent self-harm remains limited and further evaluation is needed. The Community Outpatient Psychological Engagement for Self-harm Service (COPESS) belongs to this category of interventions. It is a low-intensity therapy, which addresses causes that precipitate self-harm and associated symptoms of depression.

COPESS is a modified version of psychodynamic-interpersonal therapy (PIT), has been evaluated in two randomised trials for self-harm [27] and used in NHS self-harm services in England [30]. PIT has undergone two modifications for the purposes of this feasibility trial. Elements of another closely linked therapy, Cognitive Analytic Therapy (CAT) [31], have been added to the intervention with the use of visual mapping and a focus on identifying “exits” or solutions to clients’ difficulties. Previous evaluation of this therapy approach within an ED setting found that 64% of referred individuals attended at least one therapy session, with nearly half (*n* = 26, 49%) attending all four sessions [30]. There was also evidence of a reduction in clients’ distress over the therapy period.

The Normalisation Process Theory (NPT) [31] is an action theory that identifies key characteristics and mechanisms that can help or hinder the integration of new healthcare interventions. It has been effectively used to aid intervention development as well as evaluating and understanding implementation processes themselves. In particular, NPT offers a set of conceptual tools to aid understanding of implementation as a dynamic process [31]. NPT will be used to inform this feasibility study about the potential for normalisation of integrating the COPESS intervention into community health settings.

## Aims

The aim of this study is to examine the feasibility and acceptability of delivering the COPESS intervention in a community setting, as well as to assess the feasibility and acceptability of the trial procedures themselves, with a view to future implementation in a full-scale efficacy trial. The key outcomes concern methodological, procedural and clinical uncertainties [32–34] such as estimates of recruitment and retention rates; feasibility and acceptability of data collection instruments and data collection procedures; feasibility, acceptability and safety of the intervention.

## Methods and analysis

This protocol conforms to guidelines presented in the Consolidated Standards of Reporting Trials (CONSORT) [35] 2010 statement extension for randomised feasibility studies and clinical trial protocols. A Patient Advisory Group (PAG) was set up at an early stage to provide input into all aspects of the study. The PAG includes patients, carers and people with lived-experience of self-harm and represents a diverse range of perspectives and insight. Guidance will be sought from the PAG on a regular basis to ensure the perspectives of service users are embedded throughout the life of the study and beyond. The project has been fully reviewed by a research ethics committee who have scrutinised our procedures for managing risk and distress, and Health Research Authority approval has been granted.

### Design

The study is a single-blind, randomised controlled feasibility trial with an embedded qualitative process evaluation. Participants will be randomised 1:1 to receive COPESS plus treatment-as-usual (TAU) or TAU alone.

### Eligibility criteria

**Fig. 1:**
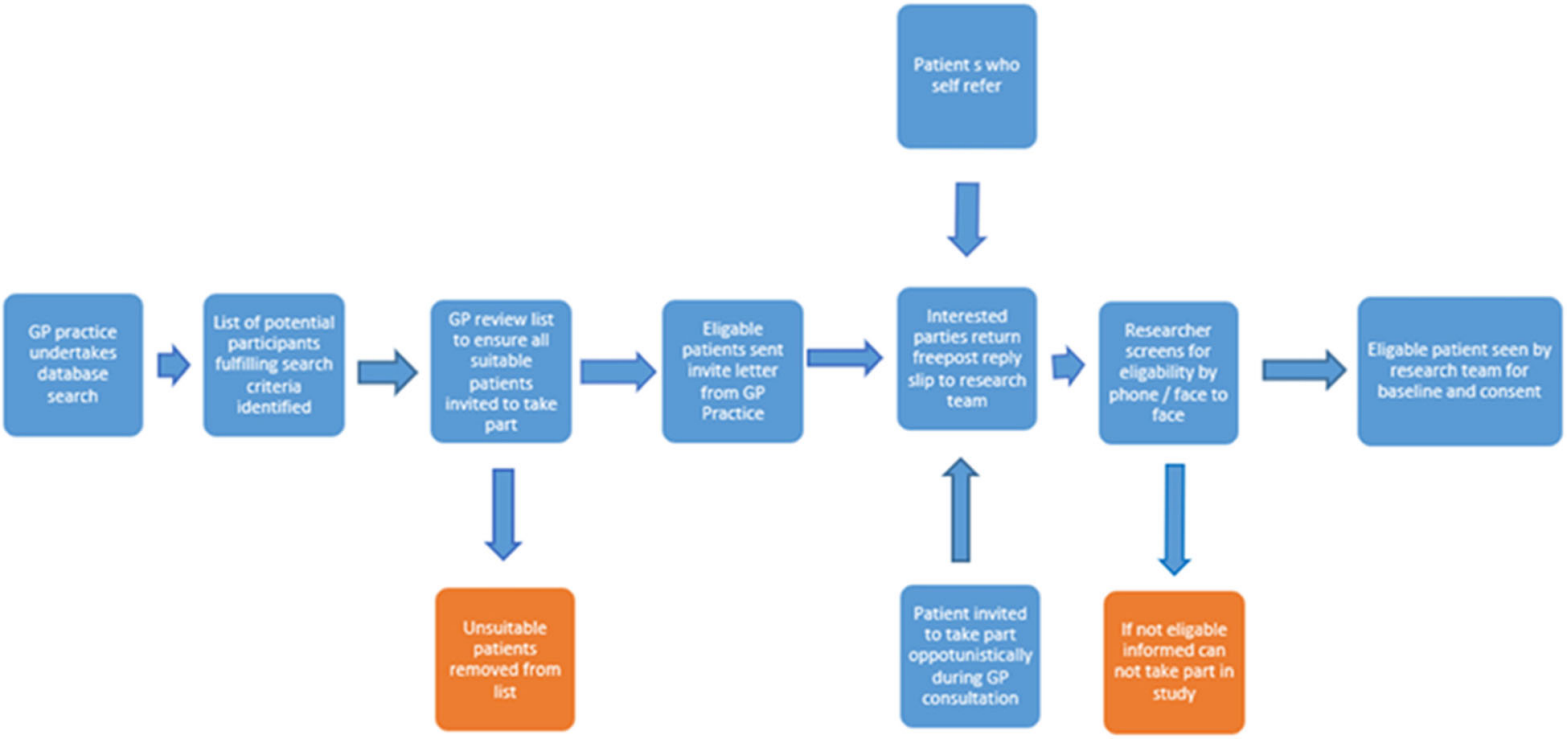
Recruitment flow chart

Participants with current depression and recent self-harm will be recruited through participating GP practices and self-referral (see Fig. 1). GPs of participants who self-refer will be informed of their participation, in line with participant consent procedures.

#### Inclusion criteria

- Adult or adolescent, aged 16 years or over.
- Self-harm within the last 6 months (self-reported) defined as an act of direct, intentional harm to oneself irrespective of suicidal intent
- A score of 14 or greater on the Beck Depression Inventory-II (BDI-II) [36]
- Help-seeking, via attendance at GP practices or self-referral into the trial.

#### Exclusion criteria

- Non-English speaking.
- Diagnosed with an intellectual disability as determined by review of clinical notes - the therapy has not yet been adapted for working with this population.
- Unable or unwilling to give written informed consent to participate.
- Currently receiving face-to-face psychological talking therapy for self-harm (potential participants will not be excluded due to group counselling or regular nurse appointments).

### Recruitment

The eligible population are adults or adolescents who self-harm and are resident in Liverpool, a large city in the North of England. There are approximately 300–350 hospital presentations for self-harm per annum to the Royal Liverpool Hospital [37]. The ‘iceberg’ model of self-harm suggests self-harm by people who present to hospital are only a small proportion of self-harm overall with most going unreported in the community [15]. Hospital figures based on routinely collected clinical data tend to underestimate self-harm presentations by around 40–50% [10]; we therefore estimate at least double the number of self-harm cases that presented to hospital emergency departments are likely to occur in the community each year [12–16].

Participants will be identified and recruited via GP practices by three methods:

1. Practice database searches with GP letters informing them of the study;
2. Participants consulting with their GP for self-harm;
3. Advertising material displayed where participants may seek help for self-harm within community settings, e.g. primary care, student counselling services, walk-in centres and Talk Liverpool (Talk Liverpool is a free NHS service that offers psychological therapies in Liverpool to adults who are feeling anxious or depressed).

### Recruitment of GP practices

The National Institute for Health Research Clinical Research Network (NIHR CRN) will assist with recruitment of GP practices. For the feasibility trial, 12 medium to large research-active GP practices will be identified and invited to participate. Initially, four recruited practices will be monitored for number of participants recruited into the feasibility trial over a 2-month period. We will add four more GP practices if required and follow the same procedure. If recruitment targets are not being met after 4 months then the final four GP practices will be included. All GPs within participating practices will be informed about the project and given an information pack (including information on ethical approval, funding and study documents) by the researcher. This may be followed up by a telephone/skype/face-to-face contact from the researcher to ascertain level of interest, and if appropriate, to arrange to discuss the study in more detail.

### Trial-GP

One GP at each practice will be identified as the (trial-specific GP) and have a portion of their time costed into the trial to make referrals, and encourage and support others at the practice to refer into the study.

### Recruitment of participants

Once potential participants have been identified and matched against the eligibility criteria, they will be sent an introductory pack that comprises: an explanatory letter from the practice; a participant information sheet about the study, an expression of interest form; and, a freepost return envelope by DocMail.

The recruitment procedure was reviewed by the PAG who believed they would be acceptable to potential participants. Although many trials rely on clinicians asking patients directly if they wish to take part we recognise this may create unwanted pressure for patients. In contrast, a letter provides greater control to the patient who then has the choice of whether to respond or not. To ensure people who self-harm are not negatively affected by receiving a letter from their GP Practice, the participant information sheet highlights details of non-NHS organisation such as Samaritans and Papyrus that patients can access regardless of whether they participate in the trial or not. Patients are informed they are free to withdraw from this study at any time, without giving a reason, and without it effecting their legal right or clinical care in any way.

Any self-referrals who contact the study team directly via community advertising methods (e.g. posters) will be asked to provide details of their GP. The study team will contact the GP to make them aware of the study and confirm patient eligibility, identify any potential risks and allow disclosures or adverse events during the study to be captured.

Recruitment rates from the different recruitment routes will be monitored, including numbers of people who do/do not respond to initial letters, proportion of eligible participants consented and the number of participants recruited against targets. Qualitative interviews with participants in the therapy arm and in the TAU arm will identify any concerns or issues relating to the study procedures. Where individuals choose not to respond to the letters and therefore do not provide consent to take part in the study or to be contacted by the study team, it is not possible to interview them or otherwise assess their experience of being contacted. However, throughout the study, adverse events and event reactions will be carefully monitored, including any adverse reactions linked to recruitment procedures. Hence, the necessary steps to monitor and identify any potential harms arising from the study will be completed throughout the feasibility trial.

### Informed consent, screening and baseline

After receiving an expression of interest from potential participants, a brief telephone/video call (e.g. Skype) screening interview with one of the research team will determine eligibility. Contact will be attempted a maximum of three times before the patient is listed as “uncontactable”. Feedback from the PAG advised that participants be given a choice of communication methods (e.g. telephone/video call/face-to-face). Those who meet the eligibility criteria will be invited for a baseline assessment. Eligibility and safety checks will be undertaken by the Research Assistant through contacting the potential participants’ GP or nurse practitioner, with the patient’s consent. Signed informed consent will be sought from all eligible participants at the initial baseline meeting.

### Reasons for non-participation and withdrawal of participation

Participants who consent to take part in the study but later decided they do not want to go forward into the trial, or leave before the intervention/TAU is complete, will be contacted and asked their reasons for not participating. For participants in the COPESS arm of the feasibility trial, therapy will continue if they choose to withdraw from the research element of the project. Where possible, data already collected will continue to be used. The reasons why participants withdrew from the study will be documented where possible. GPs will be informed of patient withdrawal and/or if a patient ceases communication with the study team**.** All feedback on non-participation or withdrawal will inform a future full trial design.

### Randomisation and blinding

**Fig. 2:**
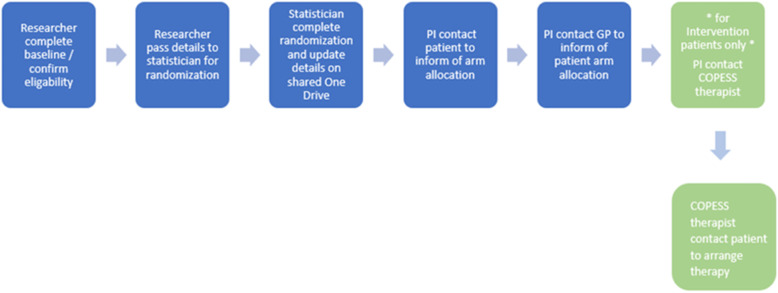
Randomization flow chart

**Fig. 3:**
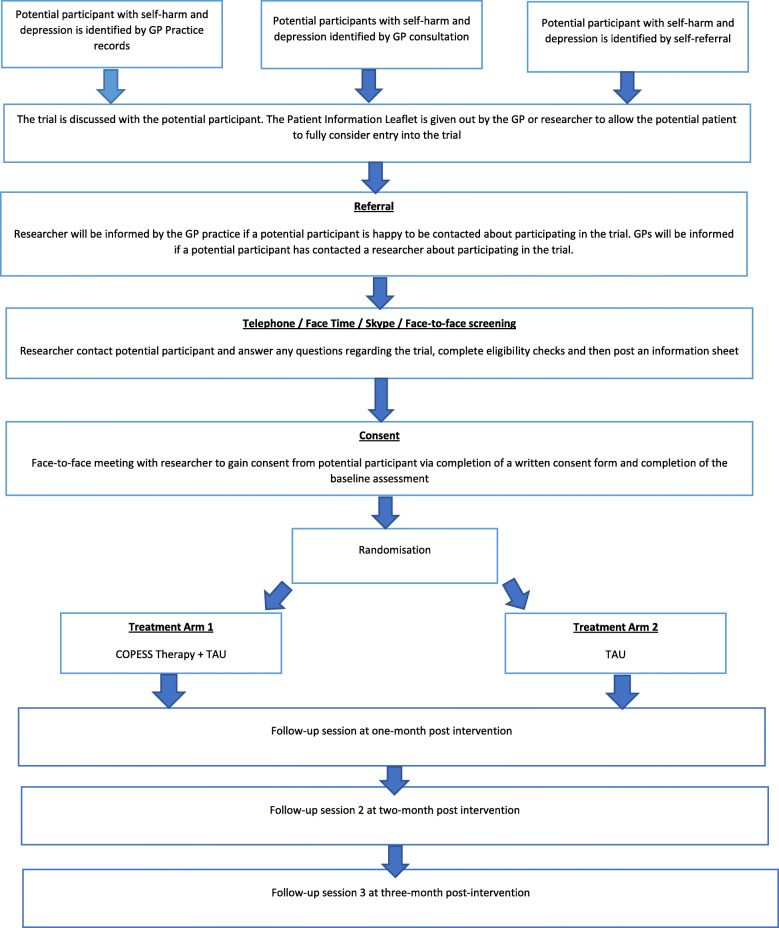
Summary of the sequence of study-related assessments, procedures and activities

Following the collection of baseline data, eligible and consenting participants will be randomly allocated (1:1) by the study statistician, to receive COPESS plus TAU or TAU alone. An algorithm within STATA 15 will be used to generate random allocation sequences in blocks of size of 4 or 6. Block sizes will occur with equal frequency and will be determined at random. The statistician generating the randomisation schedule will be independent from other elements of the project to maintain allocation concealment. Blinding is an important methodologic feature of an RCT that aims to reduce bias [34]; however, in psychotherapy trials, complete blinding is not possible as the participants cannot be blind to whether they receive therapy or not. Participants will be reminded to keep the researcher blind to their allocation arm of the study. Once randomisation has taken place the statistician will inform the study PI of patient arm allocation. The PI will inform both the patient and their GP, keeping the researcher completing study assessments masked to treatment allocation. This will be facilitated by briefing members of the research team and participants on the need to avoid disclosure of treatment details (see Fig. 2). A record will be kept of how many times accidental unblinding occurs. See Fig. 3 for full timeline of events.

### Sample size

A conventional power calculation is not necessary to achieve the stated aims of this feasibility study, as we will not be formally testing the effectiveness of the intervention. Instead, we aim to recruit 60 participants, following recommendations that this is sufficient to assess feasibility outcomes and estimate key parameters, such as the standard deviation of potential outcomes, with adequate precision in order to inform the sample size for a definitive full trial [38, 39]. For example, with *N* = 60, an attrition rate of up to 35% can be determined to within 12 absolute percentage points with 95% confidence. To avoid high attrition rates, we will keep participants engaged via text and/or telephone reminders.

### Interventions

#### COPESS plus TAU

COPESS consists of four 50-min weekly sessions of face to face or remote psychological therapy. A further follow-up session is offered 4 weeks after the end of therapy. Therapy is usually completed within 8 weeks, but variations from the expected schedule, associated reasons and drop-out rates will be monitored. Mersey Care NHS Foundation Trust will train five of their current therapists (Mental Health Nurses and Assistant Psychologists) in the COPESS therapy. The therapy involves working collaboratively with a patient to identify patterns or conflicts in emotional experiences and interpersonal relationships, linked to depressed mood and acts of self-harm. The therapist works with the patient to build a shared understanding of these experiences. The first session will focus on the participant’s most recent episode of self-harm and the interpersonal events that precipitated the episode, and the participant’s associated low mood. Risk assessment and safety planning will also be incorporated into the initial session. The three remaining sessions will focus upon the links between mood, relationship difficulties and self-harm. PIT techniques include picking up cues, staying with and exploring feelings about relationships and interpersonal problem-solving in the here and now. CAT techniques will be used to help the participant to map out and understand the reciprocal nature of relationships.

Due to the on-going impact of COVID-19 participants in the intervention arm will have the option of remote (via an online method such as Zoom or Skype or the telephone) or face-to-face therapy sessions in the participant’s home or in a community setting, once pandemic-related restrictions allow this option. There may be a difference in effectiveness of the intervention depending on whether COPESS is delivered remotely or face to face, and this will be assessed via comparison of quantitative data outcomes and through qualitative interviews**.** This choice will be solely participant preference and will not be part of the randomisation process. Safety for the therapist/researcher and/or mobility for the patient will be reviewed throughout the recruitment period.

We will calculate and present in a CONSORT [35] flow chart: the proportion of people with depression and self-harm consenting to the study; the proportion completing the baseline assessment and entering the randomised phase; the number of therapy sessions attended and the proportion completing all sessions; the proportion completing follow-up assessments at 4, 8 and 12 weeks post-randomisation.

### Treatment-as-usual (TAU)

There will be no restrictions on care provided as TAU. Participants randomised to TAU will be provided with information about how to refer to local statutory or non-statutory services and GPs will be encouraged to follow the NICE guidance on care for people who self-harm.^4^

### Therapy fidelity and adherence

COPESS will be delivered by five therapists. Therapists will receive a combined Psychodynamic Interpersonal Therapy (PIT) and Cognitive Analytic Therapy (CAT) Level 1 training. This is a 5-day short course that introduces the principles of working with PIT and CAT and the application within clinical practice. Ongoing supervision from a Lead Mental Health Practitioner will occur on a fortnightly basis. If the standard therapy approach is not being adhered to, therapists will be offered feedback. All sessions will be recorded with the consent of participants. A subset of 10% of recorded sessions will be rated by an independent psychotherapist with experience of the approach using a modified version of the Sheffield rating scale [40] to ensure adherence to the approach. Each therapist will see approximately six participants (anticipated maximum range 4–8).

### Primary outcome

The primary outcome of this feasibility study is the feasibility and acceptability of using COPESS for people in the community who self-harm and have co-existing depression. Feasibility will be defined as the ability to recruit the target sample size and retain at least 70% of participants in both arms of the trial over the 3 -month follow-up period. Acceptability of the intervention will be assessed by the percentage of participants who engage and are retained in therapy (acceptability requires > 40% randomised to COPESS to attend all sessions) with contextual data collected from semi-structured qualitative interviews focusing on participants’ experience of the therapy. Acceptability of the intervention for therapists will also be assessed by semi-structured qualitative interview. Acceptability of the trial measures will be defined as the proportion of participants who complete each of the measures at all assessment points (acceptability requires at least 80% of questionnaires completed), supplemented by semi-structured interview data.

### Secondary outcomes measures

Participants will complete a batch of standardised questionnaires at follow-up assessments (see below) to be conducted at 4, 8 and 12 weeks post-randomisation (Table 1 shows the full assessment schedule). Participants will be invited to follow-up assessments unless they have completely withdrawn from the feasibility trial. Questionnaires used for the secondary outcome measures will help to:

- Assess the feasibility of delivering these measures within an RCT context;
- Assess rates of missing data as this will inform the use of these outcomes in a full trial;
- Estimate variance in these outcomes as this will guide the power calculations for the full trial.

**Table 1:** Timing of outcome measurement

***List of questionnaire***	***Baseline***	***Follow-up 1***	***Follow-up 2***	***Follow-up 3***
*Demographic information*	*X*			
*ABUSI*	*X*	*X*	*X*	*X*
*AEP Form (A/B)*		*X*		*X*
*Beck Depression Inventory*	*X*	*X*	*X*	*X*
*CORE-10*	*X*	*X*	*X*	*X*
*ERQ*	*X*	*X*	*X*	*X*
*EQ-5D*	*X*			*X*
*HAq-II*	*X*	*X*	*X*	*X*
*SITBI 2.1*	*X*	*X*	*X*	*X*
*CSRI*				*X*

### Self-Injurious Thoughts and Behaviours Interview Short-Form (SITBI)

The SITBI is a brief interview-based measure that uniformly assesses the presence, frequency and characteristics of information on self-harm related thoughts and behaviours. Repeated self-harm during the feasibility trial period will be captured within the interview. The SITBI has demonstrated interrater reliability, test-retest reliability and convergent validity [41].

### Beck Depression Inventory-II (BDI II)

The BDI II is an established self-report measure of depressive symptoms over the past 2 weeks. There is evidence for the reliability and validity of this measure in the general population [36, 42]. Each of the 21 items on the questionnaire has a choice of four answers scored from 0 to 3. A combined score 0–13 is considered minimal depression, 14–19 mild depression, 20–28 moderate depression and 29–63 severe depression. The questionnaire takes approximately 10 min to complete.

### Frequency/Intensity of self-harm urges-Alexian Brothers Urge to Self-Injure Scale (ABUSI)

The ABUSI is a validated tool designed to evaluate the frequency and intensity of urges to self-injure over the past 7 days [43]. The scale has demonstrated good psychometric properties. The scale measures urge, regularity and strength of self-injurious thoughts across five 7-point scales. Higher scores (up to a maximum of 30) indicate a stronger desire to self-harm.

### Emotion Regulation Questionnaire (ERQ)

The ERQ is a widely validated ten item questionnaire that assesses the way in which individuals regulate their emotions, including the use of re-appraisal and suppression of emotions [44]. The scale has demonstrated good psychometric properties. Higher scores (up to a maximum of 70) indicate greater use of a particular regulation strategy.

### Clinical Outcomes in Routine Evaluation (CORE-10)

The CORE-10 is widely validated, brief ten-item measure of psychological distress over the past 7 days [45]. Higher scores signpost higher levels of psychological distress. A combined score of less than 10 falls in the non-clinical range, 11 to 14 indicates mild psychological distress, 15 to 19 moderate psychological distress, 20 to 24 moderate psychological distress and 25 or above indicates severe psychological distress.

### The Helping Relationship Questionnaire (HRQ)

The HRQ is an 11-item questionnaire that measures patient’s perception of the therapist-patient relationship [46]. The questionnaire is validated and has established psychometric properties. The questionnaire uses a six-point scale, with higher total scores indicating greater therapeutic alliance

### EQ-5D

The EQ-5D-5L is a validated six-item questionnaire measuring quality of life across five health dimensions (mobility, usual activities, self-care, pain/discomfort and anxiety/depression). Five items are measured on a five-point scale considering health that day. The final question asks individuals to signpost their health today on a 100-point scale (with zero indicating the worst health imaginable and 100 indicating the best) [47].

### The Client Service Receipt Inventory (CSRI)

The Client Service Receipt Inventory (CSRI) [48] will be used to collect healthcare resource use. This includes information on use of other primary and secondary care services, use of social services, disability payments received, personal costs related to mental health (e.g. expenditure on over-the- counter medication, expenditure on prescriptions), time off work and unpaid activities.

### Statistical analysis

As a feasibility study, we will not be carrying out hypothesis testing to determine if the intervention is effective. Data analysis will follow an Intention-To-Treat (ITT) protocol and will help inform power calculations for a future definitive COPESS trial. Attrition and reasons for drop-out will be recorded where possible. Therapists will keep records of the number of COPESS sessions participants attend and the researcher will monitor and record the same information for the data collection sessions. For participants who withdraw from the study, the researcher will follow-up with either the therapists or participants to record the reason(s) for study withdrawal. We will assess rates and types of missing data, and proportion of dropouts at different stages/pathways through the trial. We will also assess whether any of the measures display floor and/or ceiling effects. Guidance will be sought from the PAG on how to manage and minimise missing data and attrition over the study period (see earlier section further details on the role of the PAG).

Summary statistics will be used as appropriate (e.g. mean/standard deviation; median/inter-quartile range; proportion/ 95% confidence interval; data range) to describe and compare data for all participants on continuous scores for repetition of self-harm, depressive symptoms, and urges to self-harm, overall and by group. The range and standard deviation of secondary outcome measures, along with estimated attrition rate and average number recruited per practice, will be help inform sample size calculations for a future full-scale trial.

### Health economic analysis

The feasibility study will be used to develop a framework for a subsequent cost effectiveness analysis to be undertaken alongside a future RCT. In particular, economic evaluation methods will be developed and tested regarding the collection of resource use, costs and outcome data. Health care resource utilisation and absences from work will be collected for each patient during the study follow-up period using the CSRI questionnaire. Data from the feasibility study will be used to inform adaptation of the CSRI prior to a definitive full trial. Generic health-related quality of life (HRQoL) data will be collected using the EQ-5D-5L instrument.

### Qualitative study

At the time of feasibility trial consent, all participants will be asked if they are willing to be contacted for possible participation in a one-to-one interview. In-depth interviews will be conducted with participants from both arms of the feasibility trial at 4–8 weeks post-randomisation. The project adopts a comprehensive safety protocol which guides the management of risk and responses to experiences of distress. This protocol was developed jointly by researchers, clinicians and individuals with lived experience of self-harm. An experienced mental health researcher will conduct the interviews and assessments. The researcher will receive regular supervision from an experienced qualitative researcher.

We also aim to include participants who did not attend all COPESS therapy sessions. A purposive sample of participants will be interviewed to capture maximum variation in views and experience of those participating in the study. Sampling parameters will include (1) sociodemographic variables, (2) type of self-harm (injury or poisoning) and (3) feasibility trial arm allocation. Participants will be selected from both arms of the feasibility trial to provide an insight into experiences of COPESS and TAU, and to allow for comparison of these experiences. The interviews will assess understanding of, and acceptability of the intervention received (content and contexts, setting, etc.), perceived benefits and mechanisms of action, challenges to engagement and contextual factors seen to affect the impact of intervention. For participants in the TAU arm, the researcher will ask about their experience of trial participation with a focus on feasibility, study procedures and issues that may affect attrition and feasibility of a larger trial. Additionally, the researcher will explore the patient’s experience of mental health pathways within the NHS to date and whether they were aware of the COPESS intervention. Interviews will be analysed in batches and sampling will continue until no new themes emerge—we expect this to be around 16–20 interviews.

GPs at participating recruitment sites will be invited to be interviewed about their experience of recruiting participants for this study. Therapists will be invited to be interviewed about their experience of being trained in the therapy and delivering the therapy. These interviews will help gain a detailed understanding of the perceived effectiveness and acceptability of the treatment, implementation challenges, and any barriers to its uptake in a community setting. Interviews will last approximately 60 min, will be audio-recorded and transcribed verbatim. All transcripts will be anonymised.

### Qualitative data analysis

Transcripts of interview data will be analysed via Thematic Analysis using the framework approach [49]. Framework analysis was developed to meet information needs and to provide practical outcomes and recommendations [50]. It offers a highly visible and systematic approach to data analysis, showing very clearly how findings are derived. Analysis will follow the five stages of framework analysis; familiarisation with the data; identifying a thematic framework; indexing the data; charting the data; and mapping and interpretation [51]. To monitor and limit the possible bias of a single-analyst perspective, additional members of the research team with experience in qualitative methods will examine a sample of transcripts to compare their perceptions of the interview data and analysis with the main analyst’s interpretation. Themes will be discussed and refined further in multidisciplinary research team meetings.

NPT [31] provides a framework for understanding the barriers and facilitating processes that underlie the implementation and integration of complex interventions into healthcare systems. This will help make sense of the qualitative data and draw conclusions relating to how readily COPESS might be implemented and embedded into health care systems [31, 51]. The theory identifies four key processes that underlie the adoption of new interventions; coherence of intervention; cognitive participation; collective action; and reflexive monitoring. Previous research has shown that NPT can be applied effectively to qualitative data in healthcare contexts to aid interpretation [52]. NPT will be used to map the links between qualitative themes and the core processes outlined in NPT. This process will be aided through use of the NPT toolkit (normalizationprocess.org) and application of the NPT statements generated by May and colleagues [51]. To further promote integrity and rigor during the data analysis process, field notes will be written immediately after interviews and a reflective diary maintained. Thus, aiming to reduce the potential for the researcher’s values, beliefs and preconceptions to influence subsequent findings [31, 52].

### Data handling

All confidential data will be stored securely on the University research centre site with strictly limited access. Participants will be allocated an ID code which will be used on documents such as questionnaires to maintain confidentiality and minimise the use of personal data. The feasibility trial Sponsor is Liverpool John Moores University who takes primary responsibility for ensuring that the design of the study meets appropriate standards in accordance with Good Clinical Practice (GCP) guidelines. All data will be handled according to the General Data Protection Regulation (GDPR) 2018. It was agreed by the Project Steering Group that a Data Monitoring Committee was not required due to the research being a feasibility trial.

### Safety monitoring

Adverse events and risk standardised operating procedures will be developed and will be followed by all researchers and therapists working on the feasibility trial. Adverse events are defined as significant negative episodes, or significant deterioration in condition, which happen to participants during their time in the feasibility trial. These will be reported by research assistants and trial therapists to senior trial staff, who will ascertain whether these are thought to be linked to participation in the feasibility trial, and keep records of each event on an adverse events database. The Adverse Experiences in Psychotherapy (AEP) self-report measure [53] will identify adverse experiences liable to occur within psychological therapy. All serious adverse events (SAE) of an unexpected and unrelated nature will be reported to the main Research Ethics Committee, the study Sponsor and Trial Steering Committee (TSC). Suicide risk will be closely monitored. If the individual is considered to be high risk, the participant’s GP will be contacted and information passed to them within two working days. The participant will also be given advice about local crisis teams, and other relevant support services. In cases where SAE are potentially linked to the feasibility trial, withdrawal of participants, halting or terminating the feasibility trial will be considered as required.

## Discussion

The main purpose of this study is to examine the feasibility (e.g. can we recruit) and acceptability (e.g. attendance) of delivering COPESS in a community setting, and the study procedures for a future full trial. The feasibility trial will offer insight into cost-effectiveness of delivering COPESS, a brief psychological therapy, within community settings; a need that has been identified by clinicians and researchers. Positive will help support the development and a future full efficacy trial to assess how effective the intervention is at reducing depression and self-harm.

There will also be benefits beyond the immediate feasibility trial results. If COPESS proves to be an effective intervention then this new model of care has the potential to be delivered more widely within the National Health Service, as an effective, low cost, convenient, safe and easily deliverable intervention.
